# Myeloperoxidase creates a permissive microenvironmental niche for the progression of multiple myeloma

**DOI:** 10.1111/bjh.19102

**Published:** 2023-09-12

**Authors:** Connor M. D. Williams, Jacqueline E. Noll, Alanah L. Bradey, Jvaughn Duggan, Vicki J. Wilczek, Makutiro G. Masavuli, Branka Grubor‐Bauk, Romana A. Panagopoulos, Duncan R. Hewett, Krzysztof M. Mrozik, Andrew C. W. Zannettino, Kate Vandyke, Vasilios Panagopoulos

**Affiliations:** ^1^ Myeloma Research Laboratory, Faculty of Health and Medical Sciences, School of Biomedicine University of Adelaide Adelaide Australia; ^2^ Solid Tumour Program, Precision Cancer Medicine Theme South Australian Health and Medical Research Institute Adelaide Australia; ^3^ Viral Immunology Group, Discipline of Surgery, Basil Hetzel Institute for Translational Health Research University of Adelaide Adelaide Australia; ^4^ Breast Cancer Research Unit, Discipline of Surgery, Basil Hetzel Institute for Translational Health Research University of Adelaide Adelaide Australia

**Keywords:** immune suppression, multiple myeloma, myeloid‐derived suppressor cells, myeloperoxidase

## Abstract

Expression of myeloperoxidase (MPO), a key inflammatory enzyme restricted to myeloid cells, is negatively associated with the development of solid tumours. Activated myeloid cell populations are increased in multiple myeloma (MM); however, the functional consequences of myeloid‐derived MPO within the myeloma microenvironment are unknown. Here, the role of MPO in MM pathogenesis was investigated, and the capacity for pharmacological inhibition of MPO to impede MM progression was evaluated. In the 5TGM1‐KaLwRij mouse model of myeloma, the early stages of tumour development were associated with an increase in CD11b^+^ myeloid cell populations and an increase in *Mpo* expression within the bone marrow (BM). Interestingly, MM tumour cell homing was increased towards sites of elevated myeloid cell numbers and MPO activity within the BM. Mechanistically, MPO induced the expression of key MM growth factors, resulting in tumour cell proliferation and suppressed cytotoxic T‐cell activity. Notably, tumour growth studies in mice treated with a small‐molecule irreversible inhibitor of MPO (4‐ABAH) demonstrated a significant reduction in overall MM tumour burden. Taken together, our data demonstrate that MPO contributes to MM tumour growth, and that MPO‐specific inhibitors may provide a new therapeutic strategy to limit MM disease progression.

## Graphical abstract

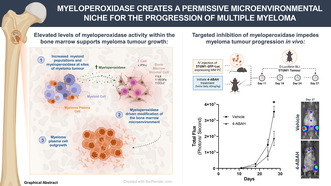

Myeloma tumour development is accompanied by an increase in myeloid cell‐derived myeloperoxidase (MPO). MPO modifies the bone marrow microenvironment with increases in IL6, VEGFa and CCL2 stromal expression, and a reduction in IFN‐γ positive cytotoxic T‐cells, providing a supportive niche for myeloma plasma cell growth. Importantly, the therapeutic targeting of MPO with the irreversible inhibitor 4‐ABAH, impedes myeloma disease progression in vivo.

## INTRODUCTION

Multiple myeloma (MM) is an aggressive haematological malignancy characterised by the uncontrolled proliferation of plasma cells (PCs) within the bone marrow (BM). Despite recent advances in therapeutic strategies, patients will ultimately relapse, with only 50% of patients surviving 5 years following diagnosis.^1^, ^2^ While intrinsic genetic factors are known to play a key role in MM pathogenesis, recent studies have highlighted the critical role of the BM microenvironment in regulating immune evasion, disease progression and persistence.^3^, ^4^, ^5^, ^6^, ^7^

The BM niche is rich in cellular components that support the survival and proliferation of malignant PCs.^8^, ^9^, ^10^ Studies have identified chronic inflammation and dysregulation of the immune environment within the tumour microenvironment as supportive of cancer development and progression.^11^, ^12^ Immunosuppressive myeloid‐derived suppressor cells (MDSCs; a heterogeneous population of immature myeloid cells) have emerged as negative regulators of disease progression in advanced MM disease.^13^, ^14^, ^15^, ^16^, ^17^ Both monocytic and granulocytic MDSCs accumulate in the BM of MM patients and tumour‐bearing mice, with increased levels correlating with disease stage and poor prognosis.^14^, ^15^ A notable hallmark of MDSC‐dependent immunosuppression in cancer is the production of reactive oxygen species (ROS), which leads to impeded CD8+ T‐cell proliferation^18^ and interferon (IFN) responsiveness.^19^ However, the precise mechanisms by which these inflammatory myeloid cells support the growth of MM remain to be determined.

Myeloperoxidase (MPO), a well‐characterised, neutrophil‐derived haem‐containing peroxidase, produces strong reactive oxidants contributing to its antimicrobial action.^16^, ^17^ We have previously reported new biological roles for MPO as a key regulator of fibrosis and angiogenesis, which contribute to cancer development and progression.^20^, ^21^ Furthermore, increased MPO activity is reported to be associated with early‐stage development of lung and breast cancer and drives tumour metastasis.^20^, ^22^ In haematological malignancies, MPO is reported to have prognostic value in B‐cell acute lymphoblastic leukaemia, with elevated expression being associated with relapse and poor event‐free survival.^23^ Notably, in a preclinical model of lymphoma, *Mpo* mRNA was found to be expressed over 57‐fold higher in granulocytic MDSCs when compared with normal neutrophils isolated from tumour‐bearing or naive mice, respectively,^24^ suggesting that granulocytic MDSCs are a rich source of MPO in cancer. However, a causal role for MPO in haematological malignancies has not yet been described.

In this study, we show increased myeloid cell‐derived MPO is associated with MM development in vivo. Specifically, elevated levels of localised MPO activity within the BM supports MM PC homing and MM tumour growth. Furthermore, MPO upregulates the expression of key MM‐supportive genes in BM stromal cells and reduces tumour‐specific T‐cell activity in vitro. Importantly, targeting MPO with the potent irreversible inhibitor, 4‐aminobenzoic acid hydrazide (4‐ABAH), significantly reduced MM tumour burden in the 5TGM1‐KaLwRij mouse model of MM. Taken together, these findings provide proof‐of‐concept data that MPO contributes to MM development and is a viable therapeutic target for the treatment of MM.

## MATERIALS AND METHODS

### 5TGM1‐KaLwRij mouse model of myeloma

The use of animals was approved by the South Australian Health and Medical Research Institute (SAHMRI) Animal Ethics Committee (SAM‐20‐022). Mice were bred and housed within the SAHMRI Bioresources Facility (Adelaide, Australia) under specific pathogen‐free conditions. Experiments were performed in accordance with the Australian Code for the Care and Use of Animals for Scientific Purposes. For tumour progression models, 8‐week‐old C57BL/KaLwRij mice were inoculated with 5TGM1 cells expressing both green fluorescent protein (GFP) and luciferase. For intravenous (i.v.) models, 5 × 10^5^ 5TGM1 cells were injected in 100 μL of PBS via the tail vein. For intratibial (i.t.) models, 1 × 10^5^ 5TGM1 cells were injected in 10 μL of PBS into the medullary cavity of the tibia as previously described.^5^, ^25^ Tumour progression was monitored and quantified by weekly bioluminescent imaging (BLI) using the Xenogen IVIS® Spectrum Imaging System as previously described.^5^ For end‐point quantification of paraprotein, serum was collected and subjected to serum protein electrophoresis (SPEP) using a Sebia Hydragel β1/β2 kit as per manufacturer's instructions (Sebia, USA). For homing models, induction of an acute inflammatory response was achieved via insertion of a 26‐gauge needle into the medullary cavity of 6‐week‐old KaLwRij mice tibiae. After 24 h, 5 × 10^6^ 5TGM1 cells in 100 μL of PBS were injected intravenously via the tail vein. The presence of GFP^+^ 5TGM1 cells after 24 h was determined using the LSRFortessa X‐20 flow cytometer. Data were analysed using FlowJoTM v10.8 Software (BD Biosciences).

### BLI of MPO activity in vivo

Mice were intravenously injected with 200 μL of luminol (30 mg/mL) at a dose of 300 mg/kg (Sigma Aldrich). After 15 min, bioluminescence was detected using the Xenogen IVIS® Spectrum Imaging System as previously described.^26^

### Targeted inhibition of MPO in vivo

4‐ABAH (Sigma Aldrich) was administered twice daily (40 mg/kg; intraperitoneal [i.p.]), in 250 μL 2% DMSO in PBS, initiated 24 h prior to tumour engraftment.

### Characterisation of BM CD11b ^+^ populations by flow cytometry

Total BM was collected from naïve or 5TGM1 tumour‐bearing KaLwRij mice at experimental end‐points, and 1 × 10^6^ cells were stained with mouse monoclonal anti‐CD11b APC‐Cy7 (BD Biosciences), anti‐Ly‐6C BV421 and anti‐Ly‐6G PE‐Cy7 antibodies (Biolegend) and subjected to analysis by flow cytometry using the LSRFortessa X‐20 flow cytometer (BD Biosciences).

### CD11b ^+^ myeloid cell isolation

CD11b^+^ cells were isolated from total BM by magnetically activated cell sorting (MACS) using the mouse CD11b Cell Isolation Kit (Miltenyi Biotec), as per manufacturer's instructions.

### Cell culture

Unless otherwise stated, cell culture media and additives were sourced from Sigma Aldrich. Cell lines (5TGM1, OP9, RPMI8226 and MDA‐MB‐231‐TXSA) were maintained as previously described.^5^, ^27^

### In vitro CD11b ^+^ assay

5TGM1 conditioned media (CM) was generated by culturing 5TGM1 (5 × 10^6^ cells/mL) in 10% fetal calf serum (FCS) (HyClone) Iscove's modified Dulbecco's medium (IMDM), supplemented with 50 μM 2‐mercaptoethanol (Sigma Aldrich). After 48 h, supernatant was collected and filtered (40 μm). CM were then collected and mixed at a 50:50 ratio with 10% FCS IMDM supplemented with 2 μM 2‐mercaptoethanol. Enriched CD11b^+^ myeloid cells were seeded (1 × 10^6^ cells/well) in CM in a 12‐well plate and cultured for 72 h.

### Conditioned media proliferation assay

Murine OP9 stromal cells were grown to 70% confluency in a T25‐cell culture flask in 10% FCS Dulbecco's modified Eagle medium (DMEM). Cells were stimulated with native human MPO (hMPO) (Cell Sciences Inc.) at increasing concentrations (vehicle, 0.5, 1, 1.5 and 2 μg/mL) in 2 mL of 1% FCS IMDM for 48 h. CM was then collected and 1 × 10^4^ 5TGM1 cells were seeded into 96‐well black/clear bottom plates (Corning Life Science) in 2% FCS IMDM with 50% OP9 CM or 2% FCS IMDM supplemented with recombinant interleukin 6 (IL6) 2 ng/mL (Sigma Aldrich) and cultured for 72 h. 5TGM1 proliferation was determined by BLI using the Xenogen IVIS® Spectrum Imaging System as previously described.^28^

### Quantitative reverse transcription polymerase chain reaction (RT‐qPCR)

Total RNA was extracted using TRIzol (Invitrogen) as per manufacturer's instructions. cDNA was synthesised using Superscript IV (Invitrogen) as per manufacturer's instructions. Quantification of gene expression was achieved using RT^2^ SYBR® Green reagent (Qiagen), on a QuantStudio™ 3 real‐time PCR system (Applied Biosystems) as previously described.^29^ Sequences for mouse primers are outlined in Table S1. Changes in gene expression were calculated relative to *Gapdh* using the ΔCt method (2^−ΔCt^).

### IFN‐γ enzyme‐linked immunosorbent spot (ELISpot) assay

Spleens collected from naive 8‐week‐old KaLwRij mice were homogenised through a 70‐μm filter and subjected to hypotonic red blood cell (RBC) lysis. Splenocytes were treated with hMPO (2 μg/mL) in 20% FCS IMDM for 20 min then cultured at 2 × 10^5^ cells/well in multiscreen‐IP HTS plates (Millipore) coated with mouse monoclonal anti‐IFN‐γ (Cat# R4‐6A2, MabTech, Sweden) with 1 × 10^5^ 5TGM1 cells for 36 h. Detection of IFN‐γ was achieved through the use of mouse monoclonal anti‐IFN‐γ (Cat#AN‐18, MabTech), streptavidin‐alkaline phosphatase and SigmaFast™ (Sigma Aldrich). Spot quantification was completed using an ELISpot reader (MabTech).

Expanded human Vγ9Vδ2 T‐cells were provided by Prof Andreas Evdokiou (Basil Hetzel Institute, The University of Adelaide).^30^ T‐cells were then treated for 2 h with hMPO in 10% heat‐inactivated FCS DMEM prior to being cultured at 1 × 10^4^ cells/well in multiscreen‐IP HTS plates coated with human monoclonal anti‐IFN‐γ (Cat#1‐D1K, MabTech) with either RPMI‐8226 cells or MDA‐MB‐231‐TXSA cells at 1 × 10^3^ cells/well for 16 h. Detection of IFN‐γ was achieved through the use of human monoclonal anti‐IFN‐γ (Cat#7‐B6‐1, MabTech) and developed as outlined above.

### T‐cell cytotoxicity

RBC depleted naïve KaLwRij splenocytes were enriched using a CD8a+ T‐cell isolation kit (Miltenyi Biotec), as per manufacturer's instructions. Murine T cells were then cocultured with gamma‐irradiated (30 Gy) 5TGM1 cells at 10:1 effector:target ratio in T‐cell medium (10% FCS RPMI‐1640 medium, 0.05 mM β‐mercaptoethanol, 1× non‐essential amino acids and 2 ng/mL recombinant hIL‐2 (R&D Systems), stimulated with or without hMPO (2 μg/mL). After 72 h, T cells were harvested and seeded at 3 × 10^4^ cells/well with 1 × 10^4^ irradiated 5TGM1 cells in 1% FCS T‐cell medium in a 96‐well plate and cultured for 24 h. Cytotoxicity was determined using a lactate dehydrogenase (LDH) assay kit (Promega Corporation) as per manufacturer's instructions.

For human T‐cell cytotoxicity assays, Vγ9Vδ2 T cells were cultured with or without hMPO (2 μg/mL) for 2 h in 10% heat‐inactivated FCS DMEM. T‐cells were then seeded at 1 × 10^5^/well in a 96‐well plate with 1 × 10^4^ RPMI‐8226 cells and cultured for 4 h. RPMI‐8226 LDH release was determined as outlined above.

### Statistical analysis

Experimental groups were compared using *t*‐test, one‐way or two‐way analysis of variance (ANOVA) as appropriate. *p*‐values of <0.05 were considered statistically significant. All statistical analyses were conducted using GraphPad Prism (version 9.0, GraphPad Software Inc.).

## RESULTS

### Myeloma tumour development is accompanied by an increase in myeloid cell‐derived *Mpo* expression in vivo

**FIGURE 1:**
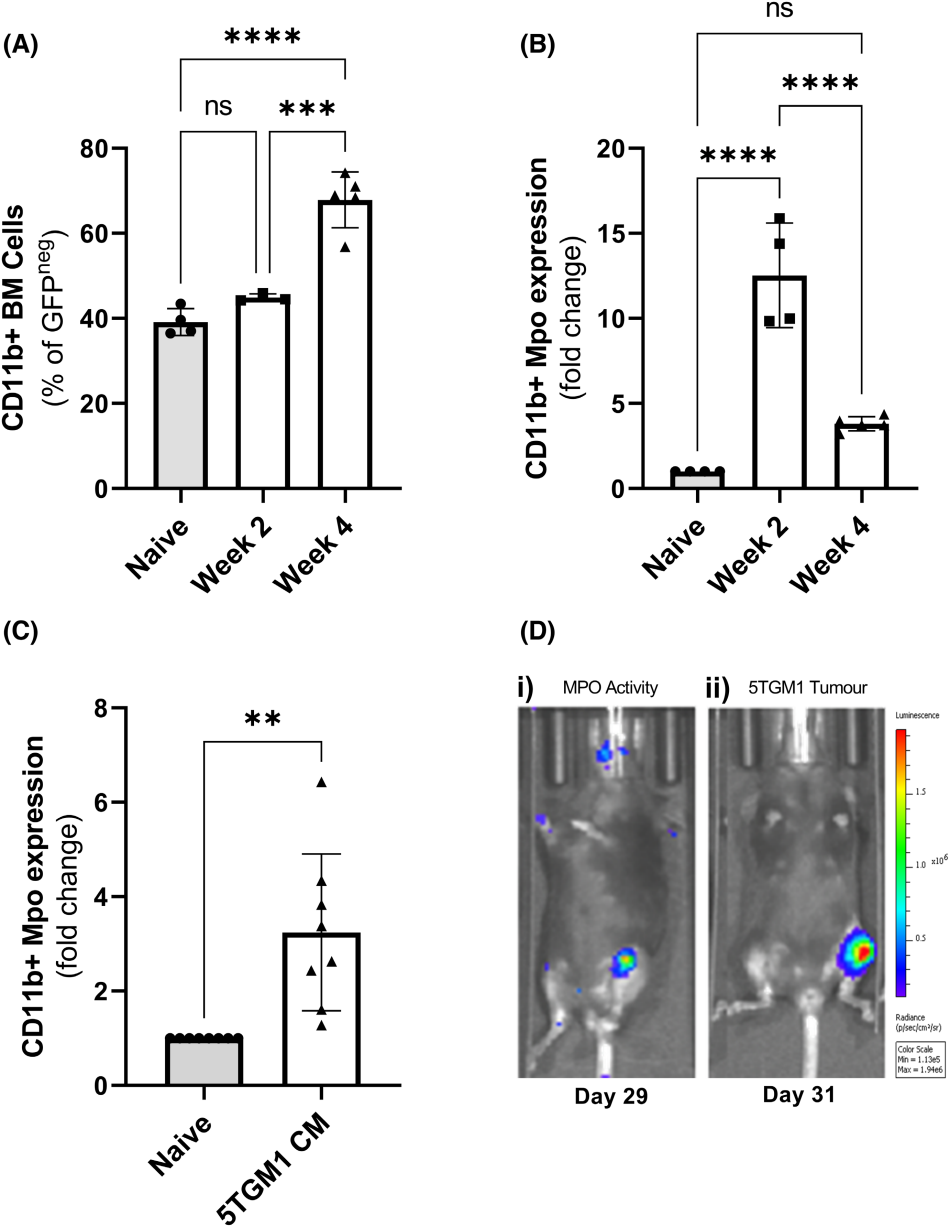
CD11b^+^ myeloid populations are expanded in the bone marrow (BM) of multiple myeloma (MM) tumour‐bearing mice, with an increase in *Mpo* mRNA expression. (A) The proportion of CD11b^+^ myeloid cells within the BM of naïve and i.v. 5TGM1 tumour‐bearing KaLwRij mice, at early (Week 2) and late (Week 4) stage tumour development, were determined by flow cytometry, represented as a % of non‐tumour (GFP^neg^) BM cells. (B) *Mpo* mRNA expression in magnetically activated cell sorting (MACS)‐enriched CD11b^+^ cells isolated from BM of naïve or tumour‐bearing (weeks 2 and 4) mice, normalised to *Gapdh*. (C) *Mpo* mRNA expression in CD11b^+^ cells isolated from tumour I mice cultured in the presence or absence of 50% 5TGM1 CM for 72 h. (D) Representative bioluminescent images (*n* = 3) from an i.t. tumour model indicating (i) myeloperoxidase (MPO) activity (luminol) colocalised at (ii) sites of MM tumour development (D‐luciferin). Results are shown as the mean ± SEM, *n* = 3–8 mice/group. One‐way analysis of variance (ANOVA) with Tukey's multiple comparisons test (A, B) or unpaired *t*‐test (C) was used to calculate significance, **p < 0.01, ***p < 0.001, ****p < 0.0001 and ns (non‐significant; *p* > 0.05). i.v., intravenous, i.t., intratibial.

Chronic inflammation can have detrimental effects within the BM microenvironment and contribute to the pathogenesis of haematological malignancies.^31^, ^32^ Therefore, we investigated whether CD11b^+^ myeloid cell populations and *Mpo* expression and activity were associated with MM development in the 5TGM1‐KaLwRij syngeneic mouse model. Consistent with previous studies,^16^, ^33^ flow cytometric analysis of BM revealed a significant increase in the CD11b^+^ myeloid cell population in response to MM tumour development at Week 4 (Figure 1A). Notably, granulocytes (identified as CD11b^+^Ly6c^int^Ly6g^+^) were enriched within the myeloid cell population in tumour‐bearing mice (Figure S1A,B). As CD11b^+^Ly6c^int^Ly6g^+^ cells from tumour‐bearing mice have previously been reported to express significantly higher *Mpo* expression compared with those from tumour‐naive mice,^24^ we next compared the expression of *Mpo* in CD11b^+^ cells isolated from BM of MM‐bearing and naive mice. BM‐derived myeloid cell expression of *Mpo* was increased almost 12‐fold at 2 weeks and fourfold at 4 weeks post‐tumour cell inoculation (Figure 1B). In addition, CD11b^+^ cells isolated from BM of naive mice cultured ex vivo with 5TGM1 CM exhibited a significant increase in *Mpo* expression (Figure 1C), suggesting MM PC may directly influence myeloid cell expression of *Mpo*. Indeed, using luminol‐BLI to detect Mpo activity in vivo,^26^ we observed increased Mpo activity co‐localised to sites of MM development (Figure 1D). Taken together, these findings indicate that MPO mRNA expression is highly upregulated within myeloid populations at early stages of MM tumour establishment, while an expansion of myeloid cells at late stage disease is accompanied by colocalisation of MPO activity.

### Localised inflammation and increased MPO activity create a pro‐tumorigenic niche for MM PC establishment and proliferation

MM development has been reported to coincide with an inflammatory BM microenvironment.^34^, ^35^ As MPO is commonly activated in inflammatory responses, we postulated that induction of inflammation, and recruitment of myeloid cells with increased MPO activity, may provide a supportive BM microenvironment to promote MM tumour development and growth.

**FIGURE 2:**
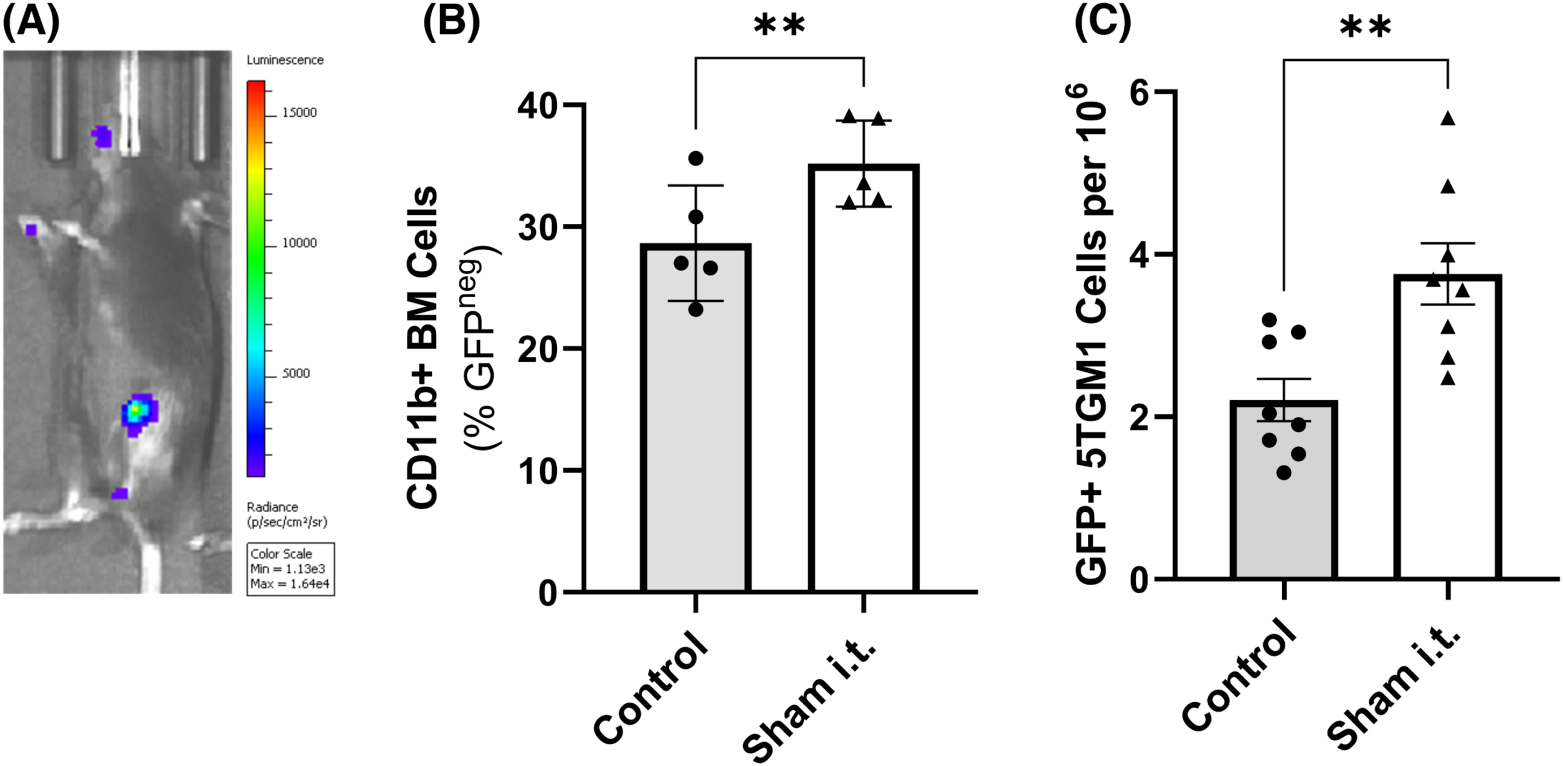
Inflammation provides a pro‐tumorigenic niche within the bone marrow (BM) and promotes multiple myeloma (MM) plasma cell (PC) homing. (A) Representative bioluminescence image indicating myeloperoxidase activity via luminol injection 24 h post‐i.t.‐induced inflammation. (B) The proportion of CD11b^+^ myeloid cells within the BM 24 h following i.t. was determined by flow cytometry. Graph shows comparison of undamaged contralateral tibia to i.t. damaged tibia in the same mouse. (C) 24 h after sham i.t. injection, an independent cohort of mice were injected with 5TGM1 MM PCs i.v. and BM of the sham i.t and contralateral tibiae were analysed after 24 h by flow cytometry to quantify the number of GFP^+^ 5TGM1 MM PCs per 10^6^ BM cells. Results are shown as the mean ± SEM, *n* = 5–8 mice/group. Paired *t*‐test was used to calculate significance, **p < 0.01. i.v., intravenous, i.t., intratibial.

To investigate this, a localised inflammatory response was induced within the BM of KaLwRij mice by insertion of a 26G needle into the i.t. medullary cavity in the left tibia. After 24 h, an increase in MPO activity was observed at the site of the sham i.t. injection (Figure 2A), accompanied by a significant increase in CD11b^+^ myeloid cells within the BM, compared with the contralateral undamaged tibiae (Figure 2B). To determine if this inflammatory BM microenvironment creates a favourable niche for MM PC homing, an independent cohort of mice were injected intravenously with 5TGM1 cells 24 h after the intratibial injury was performed. Flow cytometric analysis revealed a 1.7‐fold increase in the number of GFP^+^ 5TGM1 cells detected in the BM of the injected tibiae compared with contralateral controls (Figure 2C).

**FIGURE 3:**
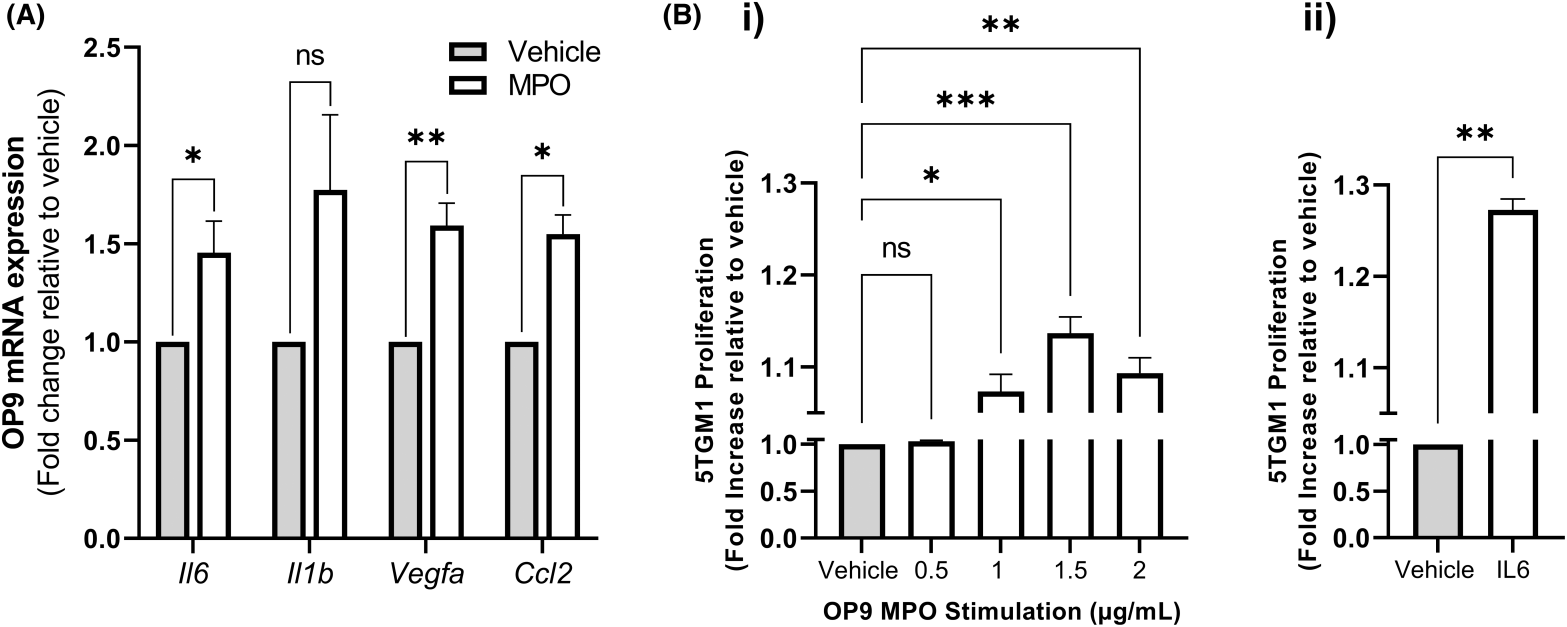
Myeloperoxidase (MPO) enhances expression of pro‐tumorigenic factors in bone marrow (BM) stromal cells (A) mRNA expression of *Il6*, *Il1b*, *Vegfa* and *Ccl2* in murine OP9 stromal cells following stimulation with 2 μg/mL MPO in vitro. (B) Proliferation of 5TGM1 multiple myeloma plasma cell cultured for 72 h in conditioned media (CM) from MPO‐stimulated OP9 stromal cells (i) or IL6 alone (ii), measured by bioluminescent imaging. Results are shown as the mean ± SEM, *n* = 3 independent experiments, performed in triplicate. Paired *t*‐test (A, B ii) or one‐way analysis of variance (ANOVA) with Tukey's multiple comparisons test (B i) was used as appropriate to calculate significance, *p < 0.05, **p < 0.01, ***p < 0.001 and ns (non‐significant) (*p* > 0.05).

We have previously demonstrated that MPO can regulate stromal cell gene expression in the context of mammary carcinoma^22^ and within the bone microenvironment.^21^ Next, we investigated if MPO can stimulate the expression of combinatorial regulators of MDSC function^36^, ^37^, ^38^ and MM development^10^, ^38^, ^39^, ^40^, ^41^ in BM stromal cells in vitro. Real‐time PCR showed that treatment with MPO increased OP9 mRNA expression of *Il6* (1.5‐fold), *Vegfa* (1.6‐fold) and *Ccl2* (1.6‐fold) compared with the vehicle control (Figure 3A). Moreover, when stimulated with CM from MPO treated‐OP9 stromal cells, 5TGM1 cells exhibited a modest but significant dose‐dependent increase in proliferation with a maximal stimulation of approximately 13% after 72 h (Figure 3B). However, stimulating 5TGM1 cells with MPO had no direct effect on proliferation (Figure S2A). Furthermore, when cultured with recombinant IL‐6 alone, 5TGM1 cells demonstrated a significant 27% increase in proliferation after 72 h (Figure 3B). Together, these data support a role for MPO in enhancing the paracrine effects of BM stromal cell‐derived factors, such as IL6, on MM PC proliferation.

### MPO suppresses T‐cell cytotoxicity in vitro

**FIGURE 4:**
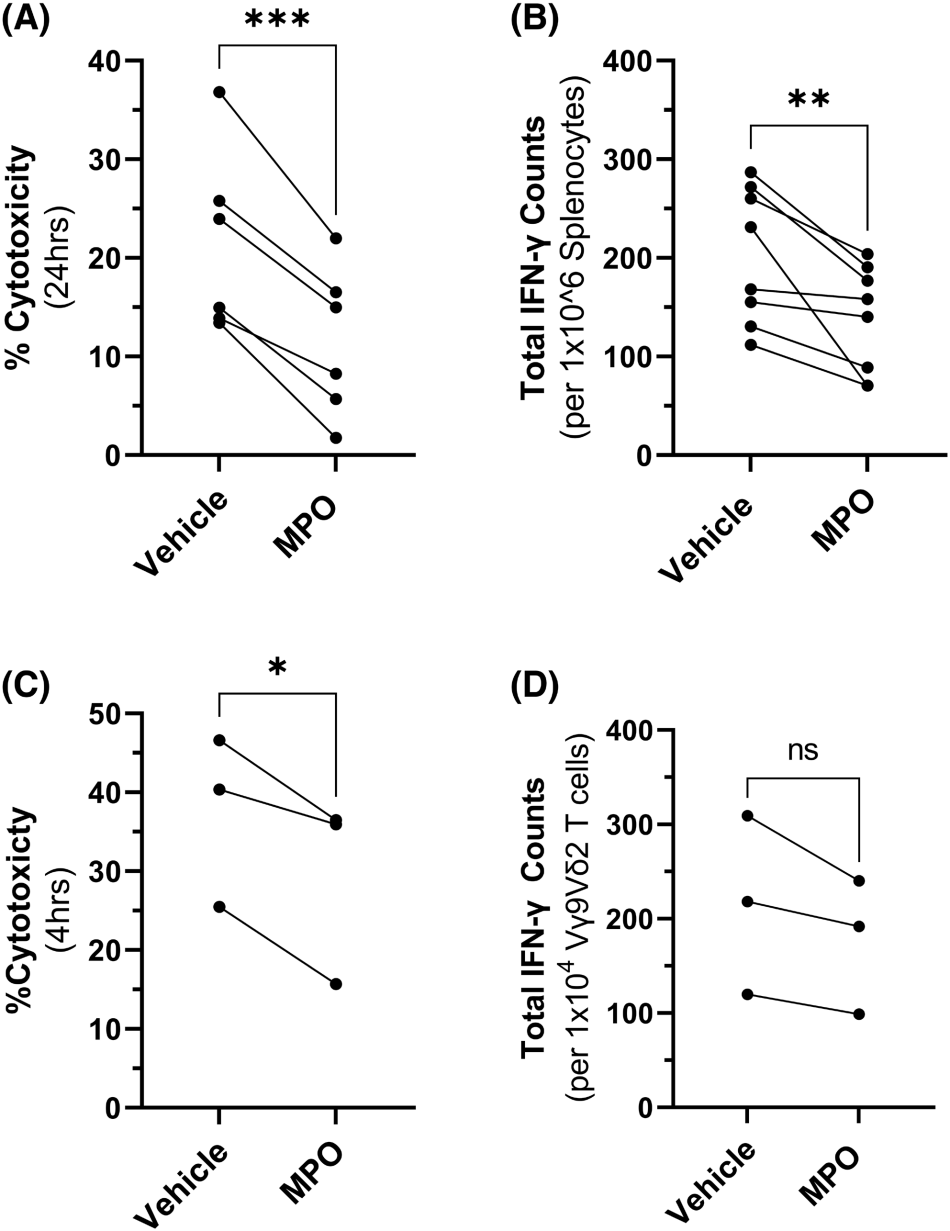
Myeloperoxidase (MPO) reduces tumour‐specific T‐cell activity. (A) CD8^+^ T‐cell cytotoxicity after 24 h (*n* = 6 mice) of culture in the presence or absence of MPO (2 μg/mL), measured by lactate dehydrogenase (LDH) release. (B) Total interferon‐γ (IFN‐γ) production by naïve KaLwRij splenocytes cocultured with murine tumour cells in the presence or absence of MPO (2 μg/mL) in an ELISpot assay (*n* = 8 mice). (C) Human Vγ9Vδ2 T‐cell cytotoxicity of human tumour cells after 4 h cultured in the presence or absence of MPO (2 μg/mL) measured by LDH release. (D) Total number of IFN‐γ‐producing cells determined by ELISpot (*n* = 3 donors). Data points represent the mean value of each donor, performed in quadruplicate. Paired *t*‐test was used to calculate significance, *p < 0.05, **p < 0.01, ***p < 0.001 and ns (non‐significant) (*p* > 0.05).

Given the early accumulation and immunosuppressive effects of MDSCs in MM disease,^16^ together with our findings that *Mpo* expression is increased during the early stages of MM development, we next aimed to investigate if MPO exerts immunosuppressive activity. CD8+ T‐cells isolated from the spleens of naïve KaLwRij mice were pretreated with MPO and their cytotoxicity towards 5TGM1 cells was assessed in vitro. Indeed, MPO treatment of resulted in a significant 46% decrease in cytotoxicity (*p* = 0.0005) compared with untreated T cells (Figure 4A). Furthermore, following MPO pretreatment, we observed a 32% decrease in IFN‐γ positive splenocytes (*p* = 0.0085), as determined using an ELISpot assay (Figure 4B). Importantly, the ability of MPO to inhibit cytotoxic T‐cell activity was confirmed using ex vivo expanded human Vγ9Vδ2 T‐cells given their potent cytotoxic capabilities. Pretreatment of Vγ9Vδ2 T‐cells with MPO resulted in a 22% reduction in cytotoxicity, compared with untreated controls, when cultured with the human MM cell line RPMI8226 (*p* = 0.048). Additionally, while there was a consistent 10%–20% reduction in IFN‐γ positive cells in all three donors, this did not reach statistical significance (*p* = 0.124; Figure 4C,D). Of note, MPO had no effect on the viability of T‐cells (Figure S3). These findings suggest that MPO may play a direct role in immunosuppression by reducing the cytotoxic potential of T‐cells.

### Targeted inhibition of MPO impedes MM tumour burden in mice

**FIGURE 5:**
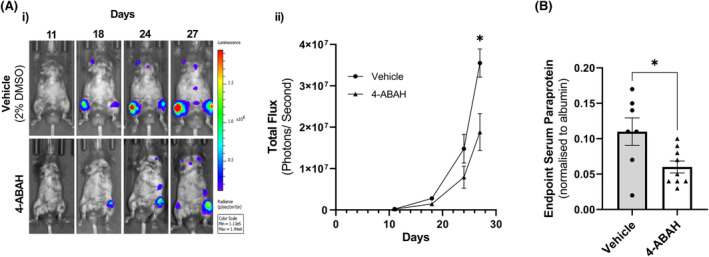
Inhibition of myeloperoxidase activity reduces tumour burden in vivo. Eight‐week‐old C57BL/KaLwRij mice were treated with 4‐ABAH (40 mg/kg; i.p.) or 2% DMSO vehicle twice daily throughout the experiment, initiated 24 h prior to inoculation of 5 × 10^5^ 5TGM1 MM PC (i.v.). (A) (i) Representative bioluminescent images; (ii) tumour burden was quantified as photons per second. (B) End‐point tumour burden was measured by serum protein electrophoresis (Day 28) normalised to internal albumin control. Results are shown as the mean ± SEM, *n* = 7–9 mice/group. Two‐way analysis of variance (ANOVA) with Šídák's multiple comparisons test (A) or unpaired *t*‐test (B) was used to calculate significance, *p < 0.05. i.p., intraperitoneal, i.v., intravenous.

Given that MPO is increased in the MM BM microenvironment in vivo and promotes MM PC proliferation and inhibits T‐cell function in vitro, the potential of specifically inhibiting MPO to limit MM tumour development in vivo was examined. KaLwRij mice were treated with the irreversible MPO inhibitor, 4‐ABAH (40 mg/kg, twice daily, i.p.) or vehicle control, beginning 1 day prior to inoculation of 5TGM1 cells. Tumour burden was assessed by BLI weekly and SPEP at experimental end‐point. Specific MPO inhibition with 4‐ABAH significantly reduced tumour burden by 48% after 4 weeks compared to vehicle at tumour end‐point (*p* = 0.041; mean BLI; vehicle, 3.55 × 10^7^ ± 3 × 10^6^ photons/s, compared with treatment with 4‐ABAH, 1.88 × 10^7^ ± 4.48 × 10^6^ photons/s; Figure 5A). These results were further supported by a significant 46% reduction in serum paraprotein levels in 4‐ABAH‐treated mice compared with vehicle controls (*p* = 0.023; Figure 5B).

## DISCUSSION

It is well established that extrinsic factors within the tumour microenvironment contribute to outgrowth and relapse of MM. The eradication of this currently incurable malignancy is dependent on understanding the complex interplay between cells of the BM microenvironment and malignant PCs, leading to their growth and survival. We have previously shown that increased MPO can produce favourable conditions for tumour progression, eliciting a pro‐fibrogenic and pro‐angiogenic tumour microenvironment in a mouse model of breast cancer.^20^, ^22^ Notably, a comprehensive meta‐analysis of 16 858 cases of genetic MPO deficiency and 21 756 control subjects revealed that lower MPO expression is associated with an overall reduced cancer risk in both solid and haematological cancers.^42^ The studies described herein identify a role for MPO in facilitating MM progression and suggest MPO as a viable therapeutic target to limit MM progression.

MDSCs accumulate within the BM of MM patients,^33^, ^34^ and *Mpo* has been established as the most highly upregulated gene within the granulocytic MDSC population in a mouse model of lymphoma.^24^ In keeping with these previous studies, we demonstrate that myeloid cell populations are increased within the BM of the 5TGM1‐KaLwRij model of MM in response to tumour development. Furthermore, our data suggest that MM PC may directly influence *Mpo* gene expression in BM‐derived myeloid cells. Specifically, we show that tumour‐associated myeloid cells exhibit a 12‐fold increase in *Mpo* expression during the early stages of tumour development, which then decreases to almost fivefold at end‐point, where we observed the highest number of MDSC accumulation. This suggests that MPO may play an important role in the early development of MM. Interestingly, a study by Van Valckenborgh et al. showed that the immunosuppressive capacity of MDSC is increased during early initiation of the myeloma tumour process.^43^ Coupled with the observation that MPO activity is co‐localised at sites of MM tumour development, these data suggest that MDSC may represent a major source of MPO in the context of MM.

The inflammatory BM microenvironment leads to myeloid skewing of haematopoietic stem cells in the context of chronic inflammation associated with ageing.^32^ In addition, myeloid cells promote pro‐inflammatory cytokine release, resulting in a vicious cycle of accumulated myeloid and myeloid‐skewed cells in the BM.^32^, ^44^ In the present study, we show that the induction of a local acute inflammatory response within the BM of KaLwRij mice resulted in the accumulation of myeloid cells and an increase in localised MPO activity. IL6, VEGFA and CCL2 are known to play a crucial role in MM tumorigenesis, both through facilitating MM PC growth and modification of the BM microenvironment.^10^, ^38^, ^39^, ^40^, ^41^ To this end, we observed a significant increase in mRNA expression of *Il6*, *Vegfa* and *Ccl2* in OP9 BM stromal cells when treated with MPO. Mechanistically, we have previously shown in stromal cells that MPO has the capacity to regulate proteins with the activation of several signalling pathways, including ERK1/2, p38 MAPK, JNK, Akt, FAK and HIF‐2a.^20^, ^45^ Furthermore, a study by Odobasic et al. showed that MPO‐derived intermediates interact with the dendritic cell complement receptor, Mac‐1 and inhibit dendritic cell activation.^46^ These findings suggest that MPO has a diverse impact on several cellular components that may modulate pro‐inflammatory/pro‐tumourigenic factors within the BM and, as such, generate a tumour‐supportive microenvironment.

Furthermore, we show an indirect effect on 5TGM1 MM PC proliferation when cultured with CM from MPO‐treated OP9 cells, which may be attributable to the upregulation of IL6. Interestingly, several case studies have detailed plasmacytoma occurrence at sites of local trauma and inflammation in MGUS patients,^47^, ^48^, ^49^ supporting the hypothesis that inflammation represents a key driving factor in the homing and/or outgrowth of malignant PCs. Collectively, these studies suggest that local trauma and the associated induction of pro‐inflammatory pathways, which we postulate include increased MPO activity, aid in establishing a favourable niche for MM development.

It is widely documented that tumour cells produce immune‐modulating factors that reprogram immature myeloid cells, namely MDSC, to acquire immunosuppressive capabilities.^50^ Furthermore, tumour progression is associated with abnormal myelopoiesis and the subsequent accumulation of MDSCs, which in turn results in the suppression of anti‐tumour immune responses.^51^, ^52^ By utilising mouse splenocytes as a rich source of murine T‐cells, in addition to ex vivo expanded human Vγ9Vδ2 T‐cells, we show that MPO pretreatment reduced both human and mouse MM PC‐specific T‐cell cytotoxicity, corresponding to a decrease in IFN‐γ‐producing cells. Interestingly, it has been previously reported that T‐cell activation and proliferation in Mpo^−/−^ mice were significantly enhanced 6 days following immunisation. Moreover, in splenocytes stimulated with ovalbumin in vitro, Mpo^−/−^ T‐cells produce higher levels of IFN‐γ compared to WT controls.^46^ Collectively, these data support a role for MPO in facilitating immune suppression by inhibiting anti‐tumour T‐cell responses.

Therapeutic targeting of MPO has recently gained considerable attention in a number of disease states, including cancer.^53^, ^54^, ^55^ Indeed, MPO inhibition resulted in a 50% reduction in tumour burden in a mouse model of lung carcinoma.^55^ While clinical manifestation of MPO deficiency has been reported to be present in approximately 1 in 4000 individuals, the majority remain asymptomatic.^56^ Therefore, although MPO is involved in normal innate immunity,^57^, ^58^ inhibiting MPO activity is unlikely to be accompanied by increased risk of infection. The potent irreversible MPO inhibitor 4‐ABAH, which functions by inhibiting the enzymatic activity of MPO, is the most extensively used MPO inhibitor in vitro, and has been used in various models of disease in vivo.^20^, ^21^, ^46^ Importantly, our study is the first to demonstrate that targeted MPO inhibition, using 4‐ABAH as a single agent, significantly reduced MM tumour burden in the 5TGM1‐KaLwRij mouse model. While encouraging, complete eradication of MM tumour was not achieved, prompting future studies utilising current anti‐myeloma therapies in combination with MPO inhibitors. Immunomodulatory drugs (IMiDs) have been well described in targeting MM tumour outgrowth through both direct cytotoxic mechanisms as well as indirectly through the modulation of tumour immunity.^59^ Notably, the immunomodulatory drug lenalidomide, commonly used in the treatment of MM, has been reported to reduce MPO levels in mouse models of colitis, and potently reduce MDSC induction in the context of MM.^60^, ^61^ Taken in conjunction with the findings presented here, this suggests a need for future studies to determine whether a combination therapy approach represents a potential means for a more pronounced reduction of MDSC‐derived MPO within the MM tumour microenvironment accompanied by a direct effect on MM PC survival, and thus a more complete response to treatment.

## CONCLUSIONS

In summary, our study has described, for the first time, that myeloid‐derived MPO contributes to MM disease progression and identifies MPO as a viable therapeutic target to reduce tumour progression in vivo. With limited therapies used in the clinic that target the stromal microenvironment, the findings presented here represent a novel treatment strategy that may be incorporated into current treatment regimens to improve the quality of life of MM patients.

## AUTHOR CONTRIBUTIONS

Vasilios Panagopoulos and Andrew C. W. Zannettino were involved in conceptualisation. Connor M. D. Williams, Jacqueline E. Noll, Makutiro G. Masavuli, Branka Grubor‐Bauk, Duncan R. Hewett and Krzysztof M. Mrozik were involved in methodology. Connor M. D. Williams, Jacqueline E. Noll, Alanah L. Bradey, Vicki J. Wilczek, Jvaughn Duggan, Makutiro G. Masavuli, Romana A. Panagopoulos and Krzysztof M. Mrozik were involved in investigation. Connor M. D. Williams and Jacqueline E. Noll were involved in writing—original draft preparation. Vasilios Panagopoulos, Andrew C. W. Zannettino, Kate Vandyke, Duncan R. Hewett and Alanah L. Bradey were involved in writing—review and editing. Vasilios Panagopoulos, Kate Vandyke, Andrew C. W. Zannettino and Jacqueline E. Noll were involved in supervision. All authors have read and agreed to the published version of the manuscript.

## FUNDING INFORMATION

This project was supported by grant 2021451 and awarded through the 2022 Priority‐driven Collaborative Cancer Research Scheme and co‐funded by Cancer Australia, Can Too Foundation and Leukaemia Foundation. J.E.N. was supported by a Veronika Sacco Clinical Cancer Research Fellowship. V.P. was supported by a National Health and Medical Research Council Early Career Fellowship. K.V. and K.M.M. were supported by Early Career Cancer Research Fellowships from Cancer Council SA's Beat Cancer Project on behalf of its donors and the State Government of South Australia through the Department of Health and Wellbeing. M.G.M. was supported by The Hospital Research Foundation Group Early‐Career Fellowship. B.G. was supported by The Hospital Research Foundation Group Mid‐Career Fellowship.

## CONFLICT OF INTEREST STATEMENT

Authors declare no conflicts of interest.

## ETHICS STATEMENT

The use of animals was approved by the South Australian Health and Medical Research Institute (SAHMRI) Animal Ethics Committee (SAM‐20‐022). Mice were bred and housed within the SAHMRI Bioresources Facility (Adelaide, Australia) under specific pathogen‐free conditions. Experiments were performed in accordance with the Australian Code for the Care and Use of Animals for Scientific Purposes.

## Supporting information


Data S1.
