# Supplementary material for: Myeloperoxidase creates a permissive microenvironmental niche for the progression of multiple myeloma
Source: Br J Haematol. 2023 Sep 12;203(4):614–24. doi: 10.1111/bjh.19102 (PMC10952523; doi:10.1111/bjh.19102)
Supplement: Supplementary file 1 — Data S1. [file BJH-203-614-s001.docx]

**Supplementary Materials:**

**Supplementary Table 1. Sequences of primers used**

| **Gene** | **Forward Sequence (5’…3’)** | **Reverse Sequence (5’…3’)** |
| --- | --- | --- |
| *Gapdh* | TGCACCACCAACTGCTTAG | GGATGCAGGGATGATGTTC |
| *Mpo* | TCCCACTCAGCAAGGTCTT | TAAGAGCAGGCAAATCCAG |
| *Il6* | GCCTTCTTGGGACTGATGCT | CTGCAAGTGCATCATCGTTGT |
| *Vegfa* | CTTGTTCAGAGCGGAGAAAGC | ACATCTGCAAGTACGTTCGTT |
| *Il1β* | GCCACCTTTTGACAGTGATGAG | AGCTTCTCCACAGCCACAAT |
| *Ccl2* | TTAAAAACCTGGATCGGAACCAA | GCATTAGCTTCAGATTTACGGGT |

**Supplementary Methods:**

**5TGM1 Proliferation Assay**

5TGM1 were seeded at a density of 1x10^4^ in 96 well black/clear bottom plates with hMPO at increasing concentrations (vehicle, 0.5 µg/mL, 1 µg/mL, 1.5 µg/mL, 2 µg/mL) in 2% FCS IMDM for 72 hours. 5TGM1 proliferation was determined by BLI.

**T Cell Viability Assay**

**Vγ9Vδ2** T cells were seeded at a density of 1x10^4^ in a 96 well format with 2 µg/mL hMPO in 10% heat inactivated FCS DMEM for 24 hours. At experimental endpoint, cells were harvested and apoptotic cells were subject to labelling for flow cytometry using a Dead Cell Apoptosis Kit with Annexin V FITC & Propidium Iodide (Invitrogen, Waltham, MA, USA) as per manufacturer’s instructions. **Cells were analysed using the BD FACSCanto II flow cytometer (BD Biosciences, San Jose, USA)** and **FlowJo v10.8 Software.**

**Supplementary Figures:**

**Supplementary Figure 1. Granulocyte and monocyte populations are expanded in the BM of tumour bearing mice.** **A)** Representative flow plots showing the gating strategy used to define granulocytic (Ly6G^+^Ly6C^int^) and monocytic (Ly6G^neg^Ly6C^+^) myeloid cell populations. **B)** The proportion of Granulocytic (i) and monocytic (ii) cells within the GFP negative (non-tumour) cell population in the BM. **C)** The ratio of granulocytes to monocytes within the total CD11b^+^ myeloid cell population. Results are shown as the mean ± SEM, n=3-5 mice/group. One-way ANOVA with Tukey's multiple comparisons test was used where indicated to calculate significance, **p<0.01, ***p<0.001 and ns (non-significant) (p>0.05).

**S****upplementary Figure 2. MPO has no direct effect on 5TGM1 proliferation**. Proliferation of **5TGM1 MM PC cultured with MPO compared to vehicle control after 72 hours, measured by BLI.** Results are shown as the mean ± SEM, n=3, performed in triplicate. One-way ANOVA with Tukey's multiple comparisons test was used to calculate significance, ns (non-significant) (p>0.05).

**Supplementary Figure 3. MPO has no effect on T cell viability.** The proportion of viable cells following 24 hours culture with 2 µg/mL human MPO or vehicle control. Results are shown as the mean ± SEM, n=2 donors, performed in triplicate. Paired t-test was used to calculate significance, ns (non-significant) (p>0.05).
